# Research on Classification Criteria for the Reducibility and Irreducibility of Intertrochanteric Femoral Fractures

**DOI:** 10.1111/os.70055

**Published:** 2025-05-03

**Authors:** Fenghua Zhu, Jinya Qiu, Liang Han, Hongxing Xu, Longtao Xiao, Qiushun Zhang, Yifeng Zhao

**Affiliations:** ^1^ Department of Traumatic Orthopedics Affiliated Hospital of Jining Medical University Jining P. R. China; ^2^ Department of Clinical Medicine Jining Medical University Jining P. R. China; ^3^ Department of Orthopedics Wenshang County Hospital of Traditional Chinese Medicine Shandong P. R. China; ^4^ Department of Orthopedics Wenshang People's Hospital Dehui P. R. China

**Keywords:** AO/OTA classification, classification criteria, femoral intertrochanteric fracture, irreducible fracture, preoperative prediction

## Abstract

**Objective:**

The current classification of intertrochanteric femoral fractures primarily follows the AO/OTA system, which guides treatment but fails to accurately predict preoperative reduction difficulty. Since reduction quality directly impacts fracture healing, internal fixation success, and patient rehabilitation, developing a classification standard that aids in predicting reduction difficulty holds significant clinical implications for achieving optimal outcomes. The purpose of this study was to develop classification criteria for femoral intertrochanteric fractures based on their reducibility and irreducibility and to provide a reference standard for preoperative predictions of the level of difficulty likely to accompany the fracture.

**Methods:**

Four hundred thirty‐seven patients with intertrochanteric fractures of the femur treated at the Affiliated Hospital of Jining Medical University and several county hospitals from January 2015 to August 2023 were reviewed. The fractures were divided into irreducible and reducible types according to actual intraoperative reduction. The imaging data were collated and analyzed, the type of fracture that may have affected the reduction was selected, the data were collated according to the type of fracture as well as the AO type, unconditional univariate logistic regression analysis was performed, and the OR values were calculated.

**Results:**

Logistic regression revealed that the risk factors leading to irreducibility were 31A3, 31A3.3, 31A1 (with obvious separation displacement), 31A2 (with anterior angular exostosis) and 31A2 (with a concomitant proximal femur fracture) fractures. Intertrochanteric fractures were typed according to the risk factors suggested by the statistical results and the specific intraoperative imaging manifestations, with irreducibility divided into 3 types and reducibility divided into 2 types, each with their respective subtypes. The accuracy of this typing in predicting the degree of difficulty of intraoperative restoration was 78.4% (343/437), and the test of consistency showed kappa = 0.573 (moderate consistency).

**Conclusion:**

Classifying intertrochanteric fractures into reducible and irreducible types can accurately preoperatively predict the difficulty of reduction for the vast majority of reducible fractures and most irreducible fractures, guide treatment, and predict the prognosis of the fracture.

## Graphical abstract

Intertrochanteric fractures were typed according to the risk factors suggested by the statistical results and the specific intraoperative imaging manifestations, with the irreducibility type divided into 3 types, the reducibility type divided into 2 types, and their respective subtypes.

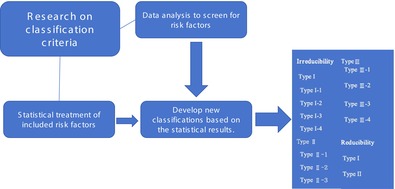

## 1 Introduction

Intertrochanteric fractures are a common consequence of trauma and occur mainly in elderly individuals. Closed reduction using traction and internal fixation with intramedullary nailing are regular treatments for these conditions [1], and most fractures can be successfully reduced by using a traction table after hip abduction, external rotation, adduction, or internal rotation [2, 3]. However, some cases of closed reduction are challenging, and a limited incision and auxiliary reduction are needed [4, 5, 6, 7, 8]. Fractures producing such challenges account for 3% to 17% of all intertrochanteric fractures [6] and are termed “irreducible intertrochanteric femoral fractures”. Currently, the AO/Orthopedic Trauma Association (AO/OTA) classification criteria are most frequently used for the clinical classification of intertrochanteric fractures, and these criteria guide treatment. However, this method has the obvious disadvantage of not providing a more accurate prediction of the difficulty of fracture reduction before surgery. Tong et al. [9] classified irreducible intertrochanteric femoral fractures into five types according to the direction of fracture displacement in the reduction process. These types included sagittal, coronal, and sagittal plus coronal, as well as two types related to the lesser and greater trochanters. However, these classification results are definitive only after surgical traction reduction and fluoroscopy. As such, they cannot guide preoperative preparations or surgical planning. In addition, preoperative decision‐makers need to be able to predict the potential difficulties of surgery to assign patients to appropriate surgeons and to ensure patient safety. Therefore, developing a set of classification criteria for intertrochanteric femoral fractures that can preoperatively predict the difficulty of fracture reduction is clinically important.

Prior to this study, we had published a classification system based on the reducibility of intertrochanteric femoral fractures in domestic journals [10]. However, in practice, this classification was confusing, difficult to remember, and difficult to promote, and it had poor application results. Additionally, the classification for reverse intertrochanteric fractures was not sufficiently detailed and was too generalized. In this study, we introduced statistical methods and used statistical tools to further verify and adjust the classification. We further refined the classification of reverse intertrochanteric fractures and discovered that this classification has many intrinsic connections with the AO/OTA classification, which were not identified in previous research. The AO/OTA classification is widely used and well known among trauma orthopedic surgeons. Therefore, based on the AO/OTA classification framework, we have made groundbreaking reconstruction and supplementation to our previous classification, achieving the goals of being easy to remember and easy to promote and having good application results. The aim was to preoperatively predict the degree of difficulty likely to be associated with femoral intertrochanteric fracture reduction and to improve the preoperative preparations and surgical scheme.

## 2 Methods

### 2.1 General Data

From January 2015 to August 2023, the Department of Traumatic Orthopedics of the Affiliated Hospital of Jining Medical University led a joint effort with the orthopedic departments of some county hospitals in Jining, Heze, and Tai'an in Shandong Province to perform 437 surgeries on patients with intertrochanteric femoral fractures. The inclusion criteria were as follows: (1) closed femoral intertrochanteric fracture; (2) age > 18 years; (3) treatment with closed reduction using traction and internal fixation with intramedullary nailing; (4) follow‐up period of at least 12 months; and (5) surgery occurred within 2 weeks of injury. The exclusion criteria were as follows: (1) surgery was performed without traction; (2) pathological intertrochanteric fractures were involved; (3) fixation was performed using bone plates; and (4) follow‐up data were incomplete or missing.

One hundred and fifty‐six (35.7%) of these patients experienced difficulties with closed reductions with traction, which were performed in conjunction with limited incisions and auxiliary reductions. We summarized and classified the clinical data from 437 patients and compared this information with previous publications [2, 3, 4, 7, 8, 9, 11, 12] to obtain classification criteria for intertrochanteric femoral fractures based on reducibility. This study was approved by the ethics committee of the Affiliated Hospital of Jining Medical University (IRB No. 2017C009). Since this study is retrospective, uses identifiable imaging data for research purposes, and does not involve personal privacy or commercial interests, the “Waiver of Informed Consent” was approved by the Medical Research Ethics Committee of the Affiliated Hospital of Jining Medical University, as detailed in the Appendix.

154 males and 283 females aged 43–102 years were included, with a mean age of 76 years. There were 227 patients with fractures on the left side and 210 patients with fractures on the right side. The causes of injury included falls (313 cases), traffic accidents (66 cases), smashes (42 cases), and falls from great heights (16 cases). The time between injury and surgery ranged from 1 to 14 days, with a mean of 3.6 days (the longer the time between injury and surgery was, the more difficult the reduction, which is why patients with more than 2 weeks between injury and surgery were excluded). The AO/OTA classifications were 31A1 in 61 patients, 31A2 in 287 patients, and 31A3 in 89 patients. Three hundred fifty‐three patients had one or more internal medical illnesses, comprising 153 cardiovascular, 126 respiratory, 161 endocrine, and 119 neurological diseases. All patients underwent a preoperative pelvic orthotopic X‐ray examination and 3D CT reconstruction to improve their preoperative assessments and to stabilize their general conditions before surgery. In this group, preoperative traction of the affected limb was not needed.

### 2.2 Surgical Methods

Intravenous inhalational anesthesia was chosen for those in good general condition, and continuous epidural anesthesia combined with subarachnoid block anesthesia was chosen for those in poor condition.

After receiving anesthesia, patients were placed supine on an orthopedic traction table for closed reduction with traction. Following traction, abduction, external rotation, adduction, and internal or external rotation of the hip were performed. A C‐arm X‐ray was used to evaluate the quality of the fracture reduction from both the force line and the displacement. The force line evaluation criteria [13] were as follows: normal or slightly valgus neck‐shaft alignment on the AP radiograph (we suggest an angle ranging from 110°–146°) [14] and an angle of anteversion of less than 20° on the lateral radiograph. The displacement evaluation criteria [15] were an AP displacement less than the medial cortical thickness and a lateral displacement less than the anterior cortical thickness. The prognosis for fracture reduction was evaluated as good if all the force lines and displacement evaluation criteria were met. If the first closed reduction did not meet the standards for good reduction, it was reduced two more times. The fractures were diagnosed as irreducible femoral intertrochanteric fractures after unsuccessful closed reduction was performed three times. Thereafter, a minimally invasive anterior clamp reduction technique [16] or limited incision prying was selected for reduction [17]. All fractures were fixed via intramedullary nailing after the procedures. Furthermore, all types could also be treated with extramedullary devices when reduction was correct [18].

### 2.3 Imaging Indicators and Application

Preoperative assessment of three‐dimensional (3D) reconstructed CT images of intertrochanteric fractures was used to estimate the extent of soft tissue damage, thereby predicting the difficulty of fracture reduction. C‐arm X‐ray imaging, including alignment (neck‐shaft angle and angle of anteversion) and degree of displacement (medial cortical displacement in the anteroposterior view and anterior cortical displacement in the lateral view), was performed intraoperatively. The intraoperative measurements and reduction quality were performed by two surgeons (one senior attending physician and one associate senior attending physician), with reference to the aforementioned alignment and displacement evaluation criteria [13, 14, 15].

### 2.4 Identification of Risk Factors Influencing Intraoperative Reduction Difficulty Based on Preoperative Imaging Characteristics

Preliminary analysis of the characteristics of the preoperative imaging data (3D reconstructed CT images) and the difficulty of intraoperative reduction revealed that most of the significantly displaced 31A1 fractures were difficult to reduce intraoperatively and that the different anteroposterior relationships between the proximal and distal fracture segments in the sagittal plane of 31A2 fractures and the presence or absence of concomitant proximal femur fractures might affect reduction. For this reason, we speculated that 31A2 fractures with the following characteristics are risk factors for difficulty in reduction: anterior angular exostosis, fragment embedding, and concomitant proximal femur fracture. We found that the original subfracture type of 31A3 fractures was correlated with the degree of difficulty in reduction, resulting in the original type being retained; in addition, we found that the type of reverse trochanteric fracture combined with a femoral neck fracture may also lead to difficulty in reduction. In summary, the following correlates that may affect reduction success were included in this study: age, sex, and AO classification and subtype (e.g., 31A1 fractures with significant displacement and 31A2 fractures with anteriorly angulated external punctures, concomitant proximal femur fractures, embedded fragments, and nonsignificant displacement).

### 2.5 Statistical Analysis

Univariate logistic regression analysis was performed using IBM SPSS 26 statistical software (version 26.0; SPSS Inc., Chicago, Illinois, USA), with irreducibility or not as the dependent variable and relevant factors affecting reset as the independent variables. The risk factor (OR) was calculated with a 95% confidence interval (*p* = 0.05). If the OR value was > 1 and *p* < 0.05, then the factor was considered a risk factor affecting reset.

### 2.6 Follow‐Up

Data on eventual patient complications collected during follow‐up and analysis of the specific distribution of these complications among patients within each fracture classification were used to determine prognostic information.

## 3 Results

### 3.1 Logistic Regression Results

The statistical results revealed that the risk factors associated with irreducibility (OR > 1, *p* < 0.05) were 31A3 (reverse oblique intertrochanteric fracture), 31A3.3, 31A1 (with obvious separation displacement), 31A2 (with anterior angular exostosis), and 31A2 (with concomitant proximal femur fracture) fractures (see Table 1).

**TABLE 1:** Univariate analyses of factors associated with irreducible fractures (*n* = 437).

Parameters	Reducible	Irreducible	*p*	95% CI	OR
(a) Demographic factors and factors based on imaging classification					
Age (< 65 y, > 65 y)	111	64	0.755	0.630–1.398	0.939
	170	92			
Gender (female, male)	182	101	0.996	0.665–1.508	1.001
	99	55			
31A2 (with anterior angular exostosis)	243	109	< 0.001	1.700–4.472	2.757
	38	47			
31A2 (with a concomitant proximal femur fracture)	278	137	< 0.001	3.739–44.176	12.852
	3	19			
31A3 (with reverse oblique intertrochanteric fracture)	244	104	< 0.001	2.040–5.329	3.297
	37	52			
31A2 (with embedded fragments)	128	143	< 0.001	0.041–0.141	0.076
	153	13			
31A1 (with obvious separation displacement)	278	131	< 0.001	5.245–59.628	17.684
	3	25			
31A1 (with slight or not displacement)	234	156	0.004	0.001–0.258	0.016
	47	0			
(b) Factors based on AO classification and subtypes					
31A1.1	260	149	0.313	0.723–2.762	1.413
	21	17			
31A1.2	273	153	0.557	0.175–2.559	0.669
	8	3			
31A1.3	274	151	0.662	0.404–4.154	1.296
	7	5			
31A2.1	162	114	0.001	0.328–0.768	0.502
	119	42			
31A2.2	238	134	0.736	0.521–1.584	0.909
	43	22			
31A2.3	235	141	0.054	0.293–1.009	0.543
	46	15			
31A3.1	270	144	0.096	0.881–4.751	2.045
	11	12			
31A3.2	278	150	0.067	0.914–15.032	3.707
	3	6			
31A3.3	258	122	<0.001	1.766–5.535	3.126
	23	34			
31A1	245	131	0.354	0.747–2.257	1.299
	36	25			
31A2	73	77	<0.001	0.239–0.544	0.36
	208	79			
31A3	244	104	<0.001	2.040–5.329	3.297
	37	52			

### 3.2 Data Analysis and Construction of Classification Criteria for the Reducibility and Irreducibility of Intertrochanteric Fractures

Statistical results revealed that there was no statistically significant association between 31A1 fractures and the ease of repositioning in the AO classification, but 31A1 fractures with significant displacement predicted irreducibility. 31A2 fractures predicted easy reducibility overall, but those with anterior angular exostosis with a concomitant proximal femoral fracture predicted irreducibility. Some 31A2 fractures with embedded fragments were irreducible, although overall for these fractures predicted reducibility. 31A3 fractures predicted irreducibility. The tentative conclusion from statistical analysis was that 31A1 and 31A2 fractures classified under the traditional AO system, which is based on the simple fracture type and the number of proximal fracture fragments, are not good predictors of the difficulty of reduction.

**FIGURE 1:**
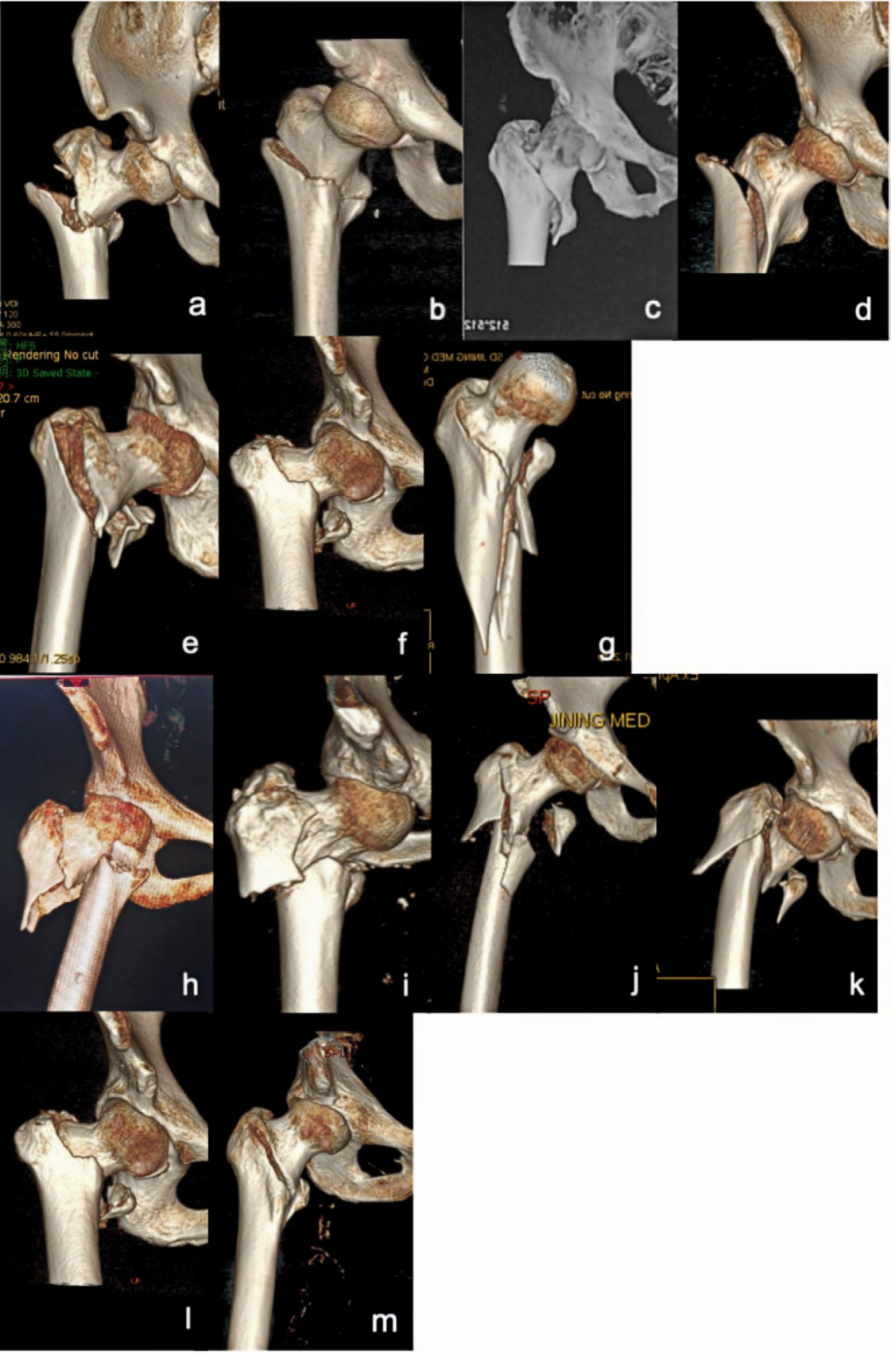
Imaging characteristics of intertrochanteric femur fractures classified based on irreducibility or reducibility. *Irreducible: (a): Type I‐1; (b): Type I‐2; (c): Type I‐3; (d): Type I‐4; (e): Type II‐1; (f): Type II‐2; (g): Type II‐3; (h): Type III‐1; (i): Type III‐2; (j): Type III‐3; (k): Type III‐4; Reducible: l: Type I; m: Type II. * Irreducible type I fractures correspond to 31A1 fractures with obvious separation displacement in the AO/OTA system, all of which are simple fractures. Irreducible type I‐1 fractures: Fracture line is just above the lesser trochanter; irreducible type I‐2: Fracture line bisects the lesser trochanter; irreducible type I‐3 fractures: Fracture line is just below the lesser trochanter and does not cut the greater or lesser trochanter; irreducible type I‐4 fractures: Fracture line extends far beyond the lesser trochanter. Irreducible type II fractures correspond to 31A2 fractures (without considering the number of intertrochanteric fracture blocks) in the AO/OTA system. Irreducible type II‐1 fractures: Proximal fracture has angular exostosis anterior to the distal fracture segment; irreducible type II‐2 fractures: Proximal fragment is embedded in the medullary cavity of the distal fracture segment, posterior to the distal fracture segment; irreducible type II‐3 fractures: Occur with a concomitant proximal femur fracture. Irreducible type III fractures correspond to 31A3 fractures (with no effect on the greater trochanter) in the AO/OTA system. Irreducible type III‐1 fractures: Simple fracture line is obliquely outward and downward; irreducible type III‐2 fractures: Simple fracture line transversely points toward the lesser trochanter; irreducible type III‐3 fractures: Occur with concomitant comminuted fracture blocks of the lesser trochanter; irreducible type III‐4 fractures: Occur with a concomitant fracture of the femoral neck. Reducible type I fractures correspond to 31A2 fractures (with the proximal fragment embedded in the medullary cavity of the distal fracture segment) of the AO system. These fractures are indistinguishable from irreducible type II‐2 fractures and account for the largest fraction of all intertrochanteric fractures. Reducible type II fractures correspond to 31A1 or 31A2 fractures (without obvious separation displacement and with mild coxa vara or coxa valga or mild alteration of the angle of anteversion).

Based on the above statistical results, we classified 31A1 fractures with obvious separation displacement as irreducible type I fractures. 31A2 fractures with anterior angular exostosis and a concomitant proximal femur fracture and some 31A2 fractures with embedded fragments were classified as irreducible type II fractures. 31A3 fractures were classified as irreducible type III fractures. Most 31A2 fractures with embedded fragments were classified as reducible type I fractures, and 31A1 fractures without obvious separation displacement were classified as reducible type II fractures. The three irreducible types of fractures were also subdivided into several subtypes based on imaging characteristics. The characteristics of each subtype are detailed in Figure 1 and Table 2.

**TABLE 2:** Classification criteria and treatment for femoral intertrochanteric fractures based on irreducibility.

Type	Imaging characteristics	Treatment*
Irreducible		
Type I	The fracture line simply dichotomizes the greater and lesser trochanters to varying degrees, and there is significant displacement between the fracture ends.	Limited incision and auxiliary reduction [9, 15, 16]
Type I‐1	Only the greater trochanter is dichotomized by the fracture line, the lesser trochanter is not affected, and the fracture line is adjacent above the lesser trochanter.	Limited incision and auxiliary reduction [9, 15, 16]
Type I‐2	Both the greater and lesser trochanters are bifurcated by the fracture line.	Limited incision and auxiliary reduction [9, 15, 16]
Type I‐3	The fracture line passes between the greater and lesser trochanters, traveling superiorly against the tip of the greater trochanter (base of the femoral neck) and passing inferiorly close below the lesser trochanter, with both the greater and lesser trochanters intact, unaffected by the fracture line, and with separation displacement between the severed ends.	Limited incision and auxiliary reduction [9, 15, 16]
Type I‐4	The fracture line dichotomizes only the greater trochanter and does not affect the lesser trochanter, extending to the subtrochanteric region.	Limited incision and auxiliary reduction [9, 15, 16]
Type II		
Type II‐1	The lesser trochanter is free, the fracture end is angled forward, the proximal fracture segment is anterior to the distal fracture segment, and the internal inferior tip of the proximal fragment embeds in the inner front side.	Limited incision and auxiliary reduction [9, 15, 16]
Type II‐2 (embedded type II)	The lesser trochanter is free, and the proximal fracture segment is embedded in the medullary cavity of the distal fracture segment with a shortened deformity.	Limited incision and pry dial reduction [4, 15]
Type II‐3	Different forms of proximal femoral fractures with concomitant intertrochanteric femoral fractures.	Limited incision and auxiliary reduction [9, 15, 16]
Type III	The main fracture line is obliquely directed outward and downward at varying degrees from above the lesser trochanter or via the lesser trochanter; the greater trochanter is usually unaffected. The partial proximal fracture segments are abducted and present different degrees of coxa adducta.	Limited incision and auxiliary reduction [9, 15, 16]
Type III‐1	The fracture line is simple and obliquely directed outward and downward from the lesser trochanter, and the greater trochanter is not affected. The lateral wall has an obvious tip and embeds outward below, with a more obvious coxa adducta.	Limited incision and auxiliary reduction [9, 15, 16]
Type III‐2	The fracture line is simple and reaches a lateral direction horizontally from the lesser trochanter, and the greater trochanter is not affected. Coxa adducta is present, partially evident and partially inconspicuous.	Limited incision and auxiliary reduction [9, 15, 16]
Type III‐3	The lesser trochanter appears to collide with the fracture block based on irreducible type I or type II fractures; it can be complicated by an additional fracture line of the lateral wall.	Limited incision and auxiliary reduction [9, 15, 16]
Type III‐4	Femoral neck fracture occurs simultaneously with irreducible type II‐1, type II‐2, or type II‐3 fractures.	Limited incision and auxiliary reduction [9, 15, 16]
Reducible		
Type I (embedded type I)	The lesser trochanter is free, and the proximal fracture segment is embedded into the medullary cavity of the distal fracture segment with a shortened deformity.	Traction reduction [1, 2, 3]
Type II	The fracture line is parallel to the intertrochanteric line, and there is no or slight separation displacement between the distal and proximal fracture segments; displacement manifests as a mild change in the collodiaphysial angle, femoral neck anteversion, or displacement in only the lesser trochanter.	Traction reduction [1, 2, 3]

* For irreducible femoral intertrochanteric fractures, traction reduction was the first choice, and a limited incision and auxiliary reduction were performed if the reduction was not ideal.

### 3.3 Detailed Introduction of the New Fracture Classification and Imaging Features of Each Classification

The AO classification emphasizes both the degree of comminution of the posterior medial cortex of the intertrochanteric fracture and whether the fracture affects the lateral cortex, focusing on predicting the stability of the intertrochanteric fracture. The instability of intertrochanteric fractures is characterized mainly by comminution of the posterior medial cortex of the proximal femur, extension of the fracture line into the subtrochanteric region, and reversal subfractures. Therefore, 31A1.1, 31A1.2, 31A1.3, and 31A2.1 fractures are stable fractures, and 31A2.2, 31A2.3, 31A3.1, 31A3.2, and 31A3.3 fractures are unstable fractures. Prediction of whether a fracture is stable or unstable can guide clinical treatment, facilitate peer‐to‐peer communication, and guide prognosis. However, our study revealed that, among the stable fractures, most 31A1 fractures with obvious separation displacement and 31A2.1 fractures with obvious anterior angular exostosis and embedded fragments located anteriorly to the anterior wall of the distal fracture segment were difficult to reduce and were prone to secondary displacement or internal fixation failure. Among the unstable fractures, most 31A2.2 fractures and 31A2.3 fractures with the proximal fragment embedded in the medullary cavity of the distal fracture segment that were located posterior to the anterior wall of the distal fracture segment were easy to reduce and were less likely to experience secondary displacement or failure of internal fixation. Therefore, we believe that 31A1 and 31A2 fractures classified under the traditional AO system cannot be used to predict the degree of difficulty of reduction or the prognosis of the fracture.

Our research also revealed that 31A2.3 and 31A3 fractures and those with significant anterior angular exostosis and embedding the proximal fragment anterior to the distal fracture's anterior cortex (31A2.1 and 31A2.2 fractures, respectively) were difficult to reduce. Below, we provide a detailed description of the imaging characteristics of each subtype within the AO classification framework for intertrochanteric fractures based on their reduction difficulty.

#### 3.3.1 Irreducible Fractures

##### 3.3.1.1 Irreducible Type I Fractures

These fractures are classified under the AO/OTA system as 31A1 fractures. The fracture line simply dichotomizes the greater and lesser trochanters to varying degrees, and there is significant displacement (≥ 1 cm at the site of maximum fracture displacement) between the fracture ends [19] (Figure 1a–d). Based on the degree to which the greater and lesser trochanters are affected, these fractures can be further divided into the following 4 subtypes: ① Irreducible type I‐1 fractures (Figure 1a): These fractures are classified under the AO/OTA system as 31A1.1 fractures. The main feature of these fractures is that the greater trochanter is bifurcated by the fracture line, which lies medially just above the lesser trochanter. The lesser trochanter is located in the distal fracture segment, with significant displacement between the fracture ends. The proximal fracture segment usually has a long tip at the anterior‐medial end. In addition, the distal fracture segment is located in the front part of the hip due to traction from the iliopsoas. On clinical examination, the anterior aspect of the hip Is clearly distended and convex. ② Irreducible type I‐2 fractures (Figure 1b): These fractures are classified under the AO/OTA system as 31A1 fractures. The main feature of these fractures is that the fracture line bifurcates the greater and lesser trochanters [20], with obvious separation displacement between the fracture ends. The proximal fracture segment is flexed and rideable, straddling the distant folding section. ③ Irreducible type I‐3 fractures (Figure 1c): These fractures are classified under the AO/OTA system as 31A1 fractures. The main feature of these fractures is that the fracture line does not affect the greater or lesser trochanter but rather extends below the lesser trochanter, with significant separation displacement between the fracture ends and significant inward and downward separation displacement of the proximal fracture segment. ④ Irreducible type I‐4 (Figure 1d): These fractures are classified under the AO/OTA system as 31–A1.3 fractures. The main feature of these fractures is that the fracture line bifurcates the greater trochanter but not the lesser trochanter, extending to the subtrochanteric region with significant separation displacement between the fracture ends.

##### 3.3.1.2 Irreducible Type II‐1 Fractures

These fractures are classified under the AO/OTA system as 31A2 fractures. The main feature of these fractures is that the proximal fracture segment (cephalocervical segment) is located slightly anterior to the distal fracture segment and has a distinct front inner lower tip. There is a distinct cleft between the broken ends, resulting in anterior (Figure 1e) angulation. The lesser trochanter is free and located on the posteromedial side of the proximal segment. Lateral X‐ray images show the proximal segment in anterior rotation so that it lies anteriorly to the distal segment. Clarifying the anterior–posterior overlap between the distal and proximal fracture segments on orthogonal X‐ray is difficult. Under intraoperative traction, proximal fractures are most often clearly rotated anteriorly. They are not significantly displaced on AP radiographs, but fissures formed by anterior angulation between the fracture ends can still be identified.

##### 3.3.1.3 Irreducible Type II‐2 Fractures

These fractures are classified under the AO/OTA system as 31A2 fractures. These fractures cannot be preoperatively distinguished from reducible type I fractures by imaging alone. All abovementioned fractures have proximal embedded fragments in distal fracture segments within the medullary cavity (embedded type II) (Figure 1f). The general procedure for treating intertrochanteric fractures involves conventional traction, followed by abduction, external rotation, adduction, and internal rotation or external rotation. When the overlap of the above procedures in a locked state does not release the fracture, the proximal fracture segment is stuck and locked on the posterior side of the anterior wall of the distal fracture segment, or the fracture remains locked after more than three repeated reductions, a fracture can be classified as irreducible type II‐1 (embedded type I)I. Otherwise, the fracture is reducible type I (embedded type I).

##### 3.3.1.4 Irreducible Type II‐3 Fractures

These fractures are classified under the AO/OTA system as 31A2.3 fractures. They are definite anterograde intertrochanteric fractures with a concomitant long segmental fracture of the proximal femur (Figure 1g).

##### 3.3.1.5 Irreducible Type III Fractures

These fractures are classified under the AO/OTA system as 31‐A3 fractures. The main fracture line is obliquely directed outward and downward to varying degrees from above the lesser trochanter or via the lesser trochanter. The greater trochanter is usually not affected, but an additional fracture line may be present, resulting in a lateral wall fracture. The lesser trochanter may be a simple or comminuted fracture (Figure 1h–k). A portion of the proximal fracture segment is abducted, and different degrees of coxa vara are present. Irreducible type III fractures are further divided into 4 subtypes based on the direction of the main fracture line, the extent of the lesser trochanter fracture, and the presence of a concomitant femoral neck fracture: ① Irreducible type III‐1 fractures (Figure 1h): The fracture line is simple, with no additional fracture blocks. The main fracture line is obliquely directed outward and downward from the lesser trochanter, and the greater trochanter is not affected. The lateral wall has an obvious tip that embeds outwardly and downward, and the coxa vara is more obvious. ② Irreducible type III‐2 fractures (Figure 1i): The fracture line is simple with no additional fracture blocks. The main fracture line reaches the lateral direction horizontally from the lesser trochanter, and the greater trochanter is not affected. Coxa vara is present, partially evident, and partially inconspicuous. ③ Irreducible type III‐3 fractures (Figure 1j): A smashed fracture block, based on irreducible type I or type II fractures, appears in the medial lesser trochanter. An additional fracture line of the lateral wall may complicate this process. ④ Irreducible type III‐4 fractures (Figure 1k): These femoral neck fractures occur simultaneously with irreducible type III‐1, III‐2, or III‐3 fractures.

#### 3.3.2 Reducible Fractures

##### 3.3.2.1 Reducible Type I Fractures

These fractures are classified under the AO/OTA system as 31A2 fractures. Specifically, the proximal fracture segment is embedded in the medullary cavity of the distal fracture segment and is located posterior to the distal fracture segment (Figure 1l). The lesser trochanter is mostly free and is located posteriorly and medially to the proximal segment. The distal‐medial end of the proximal segment also has a distinct tip.

##### 3.3.2.2 Reducible Type II Fractures

Most of these fractures are classified under the AO/OTA system as 31A1 fractures, but some are classified as 31A2 fractures (Figure 1m). The main features include slight or no separation displacement. Some patients may have a decreased angle of anteversion, a decreased neck‐shaft angle, no separation displacement between fracture ends, and separation displacement of only the lesser trochanter.

### 3.4 Evaluation of the Accuracy of Fracture Classification

Analysis of the recorded degree of difficulty of intraoperative reduction among patients within each new fracture classification indicated that the overall accuracy of the new subtyping was 78.4% (343/437) (see Table 3). The kappa consistency test yielded kappa = 0.573, and moderate consistency was obtained.

**TABLE 3:** Accuracy of the preoperative and intraoperative evaluation results for all types of fractures.

Type	Preoperative evaluation	Intraoperative evaluation	Accuracy rate (%)
Irreducible			
Type I	28	25	89.29
Type I‐1	6	3	50.00
Type I‐2	16	16	100.00
Type I‐3	1	1	100.00
Type I‐4	5	5	100.00
Type II	104	66	63.46
Type II‐1	85	47	55.29
Type II‐2*	0	13	—
Type II‐3	22	19	86.36
Type III	89	52	58.43
Type III‐1	18	10	55.56
Type III‐2	7	5	71.43
Type III‐3	44	26	56.76
Type III‐4	20	11	59.09
Reducible			
Type I	166	153	92.17
Type II	47	47	100

* Preoperative prediction is not possible.

### 3.5 Follow‐Up Results

All patients were followed up for 13–25 months (mean 17.6 months). Eighteen patients presented coxa vara (12 with irreducible type III fractures, 2 with irreducible type II‐1 fractures, 1 with an irreducible type II‐2 fracture, 1 with an irreducible type II‐3 fracture, 1 with an irreducible type I fracture, and 1 with a reducible type I fracture). Two patients had fractures with the spiral blade cut out of the femoral head (1 with an irreducible type II‐1 fracture and 1 with an irreducible type III fracture); three had fractures with partial withdrawal of the spiral blade (2 with irreducible type II‐1 fractures and 1 with a reducible type I fracture); nine had lateral wall fractures with separation displacement of the lateral wall after fixation (9 with irreducible type III fractures); and one had a lower‐limb internal rotation deformity (1 with an irreducible type III fracture). Ultimately, four patients experienced internal fixation failure (two died from infection, one required joint replacement, and one was left with traumatic arthritis); 20 patients were left with varying degrees of claudication. The remaining patients exhibited good fracture healing (on radiography) and good recovery of ambulation. The healing time ranged from 3 to 9 months, with a mean of 5.9 months (see Table 4 for details).

**TABLE 4:** Distribution of complications among patients with each type of fracture.

Type	Coxa vara	Cut out	Withdrawal of spiral blade	Redisplacement of the lateral wall	Internal rotation deformity of lower limbs
Irreducible					
Type I	1	0	0	0	0
Type II‐1	2	1*	2**	0	0
Type II‐2	1	0	0	0	0
Type II‐3	1	0	0	0	0
Type III	12	1***	0	9****	1
Reducible					
Type I	1	0	1	0	0
Type II	0	0	0	0	0

* The patient (age 91 y) was prematurely weight‐bearing one month after PFNA internal fixation, and a follow‐up visit revealed that the spiral blade had cut through the femoral head but had just penetrated. The patient was instructed to remain strictly bedridden and to avoid weight bearing. Three months after the operation, the fracture healed firmly, the spiral blade was not cut, and the patient regained his walking ability. At the time of a follow‐up telephone call 9 years later (age 100 y), the patient was still able to walk but experienced pain in the hip after walking.

** Two patients with irreducible type II‐1 fractures died from premature unprotected weight bearing after internal fixation, one of whom developed spiral blade withdrawal and one of whom experienced fracture displacement 1 month after surgery and in whom internal fixation eventually failed. The patients were advised to strictly remain in bed and avoid weight bearing. For the first patient, 1 year after surgery, the tail of the spiral blade punctured the skin, causing infection and ultimately resulting in death from infectious shock and hypostatic pneumonia. In the other patient, because of fragment embedding and displacement 1 month after surgery, the patient experienced pain after activity and died of hypostatic pneumonia due to long‐term bed rest.

*** In one patient with an irreducible type III–4 fracture, the spiral blade was cut out 4 months after surgery, the fracture did not heal, and the internal fixation failed. Finally, the half‐hip joint was replaced to restore the walking function.

**** Nine patients with irreducible type III‐3 fractures with lateral wall additional fracture lines had pronation and abduction displacement again in the lateral wall after internal fixation. The patients were advised to postpone weight bearing to protect the affected limb from trauma and to have regular outpatient reviews. All of these patients achieved bone healing.

## 4 Discussion

There is a clinical consensus on the importance of avoiding deformities during intertrochanteric fracture reduction and thereby obtaining better functional recovery [21, 22, 23, 24, 25, 26, 27, 28]. Intertrochanteric fracture reduction also reduces the probability of internal fixation failure and facilitates early functional exercise [21, 22, 23, 24, 25, 26, 27, 29]. The quality of reduction is controllable [22, 23, 24, 25, 26, 27, 30] through the accurate preoperative prediction of the degree of difficulty of reduction, optimization of preoperative preparations, and surgical planning. However, the traditional AO/OTA classification as well as the Evans classification and its improved version cannot accurately predict the difficulties associated with foundational reduction. Therefore, we formulated classification criteria for intertrochanteric fractures based on their reducibility or irreducibility. These findings are based on the imaging data of intertrochanteric femoral fractures from 437 patients combined with relevant published information.

### 4.1 Characteristics of Each Type of Fracture

Our study indicates that after an intertrochanteric fracture of the femur, the success of closed reduction with traction depends on the extent of damage to the ligamentous structures of the hip joint (including the iliofemoral, pubofemoral, and ischiofemoral ligaments) and the joint capsule. Under traction, the proximal fracture fragment tends to flex, abduct, and externally rotate due to the pull of the gluteus medius, gluteus minimus, iliopsoas, and external rotator muscles. If the hip joint ligaments and capsule remain fully or relatively intact, these soft tissue structures act as a splint, counteracting the flexion, abduction, and external rotation of the proximal fragment. Combined with longitudinal traction applied distally, this facilitates satisfactory fracture reduction. Conversely, if these structures are significantly damaged, achieving proper reduction becomes challenging. Below, we analyze each fracture type in detail.

#### 4.1.1 Irreducible Type I

Fractures account for a low percentage of irreducible intertrochanteric fractures. According to the new fracture classification criteria reported here, 28 patients had fractures of this type, 25 (89.3%) of which were difficult to reduce intraoperatively. Therefore, irreducible type I fractures should be treated with the utmost care and adequate preoperative preparation. The reason for the difficulty in reducing these fractures is associated with pull from the iliopsoas on the lesser trochanter and from the gluteus and external rotator muscles on the greater trochanter.

Irreducible type I fractures correspond to 31A1 fractures under the AO/OTA system. 31A1 fractures are stable fractures associated with mild injury and good prognosis. However, we found that 31A1 fractures with significant displacement had a poor prognosis due to heavy soft tissue hinge injury (rupture of the iliofemoral ligament and the joint capsule) and instability, leading to difficulty of intraoperative reduction. Therefore, we classified these fractures as irreducible type I fractures. 31A1 fractures with less separation displacement generally have good prognoses due to light soft tissue hinge injuries and stability and thus were easily reduced intraoperatively and classified as reducible type II fractures. These findings suggest that the new fracture classification criteria, which consider the degree of soft tissue damage, have a significant advantage over the AO/OTA system in guiding the intraoperative management of fractures.

#### 4.1.2 Irreducible Type II‐1 Fractures Versus Reducible Type I Fractures

**FIGURE 2:**
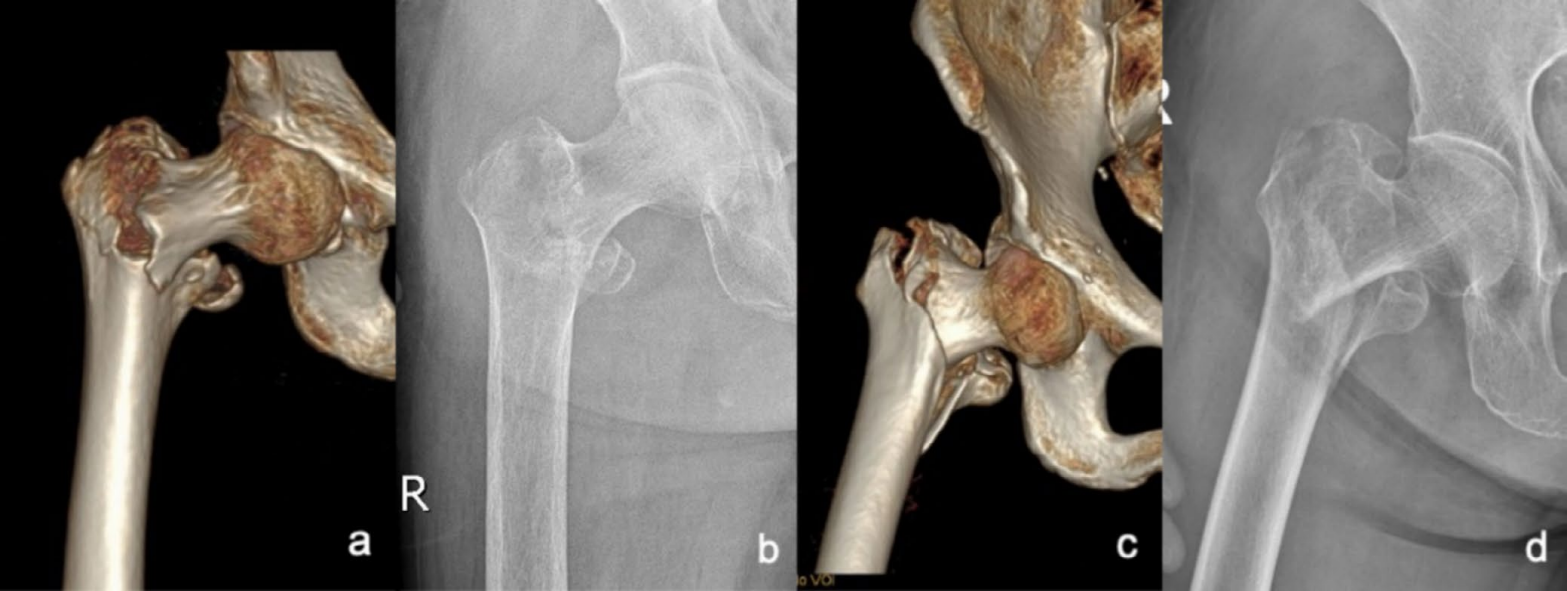
Comparison of preoperative images of irreducible type II‐1 fractures and reducible type I fractures. An irreducible type II‐1 fracture and a reducible type I fracture appear significantly different in 3D reconstructed CT images (a and c respectively) but are difficult to distinguish in anteroposterior X‐ray images (b and d, respectively).

It is difficult to distinguish between irreducible type II‐1 fractures and reducible type I fractures on preoperative X‐rays because the different shifts in the anterior and posterior directions of the proximal fracture segments cannot be distinguished (it is often difficult to identify the standard lateral tablets as a result of pain) (see Figure 2). The iliofemoral ligament and the articular capsule attached to the intertrochanteric line of reducible type I fractures remain relaxed because of the embedding of the proximal segment into the distal segment of the medullary cavity. After the overlapping displacement is corrected under traction, the iliofemoral ligament and the joint capsule (acting as a soft tissue splint) regain normal tension and prevent further flexion, abduction, and external rotation of the proximal segment, ultimately leading to good reduction. In irreducible type II‐1 fractures, the proximal fracture segment is anterior to the distal fracture segment, and the tip of the proximal fracture segment embeds the front inner lower side, resulting in rupture of the iliofemoral ligament and the joint capsule. Without bony blockage of the distal fracture segment or restriction of the soft tissue splint, the proximal fracture segment is characterized by hyperflexion, hyperabduction, or excessive external rotation under the pull of the iliopsoas, gluteus, or external rotator muscles, making reduction difficult. Therefore, these two types of fractures differ in the degree of difficulty of reduction due to differences in the degree of soft tissue injury. When these fractures are associated with partial rupture of the iliofemoral ligament or when the joint capsule and continuity remain, splinting of the remaining soft tissue under traction contributes to good reduction. No difficulties of reduction occurred in 38 (38/85) of the irreducible type II‐1 fractures in this study. Both irreducible type II‐1 and reducible type I fractures are classified as 31A2 in the AO/OTA system, but our proposed criteria consider the effect of the degree of soft tissue injury on fracture reduction.

According to the new fracture classification criteria, irreducible type II‐2 fractures cannot be accurately predicted before surgery. Their imaging presentation is similar to that of reducible type I fractures, with the proximal fracture segment embedded into the medullary cavity of the distal fracture segment and a free lesser trochanter. These fractures are therefore usually preoperatively classified as reducible type I fractures (embedded type I). They are classified as 31A2 fractures in the AO/OTA system. The most common type of intertrochanteric fracture was fractures with embedded fragments (embedded type I and embedded type II), accounting for 37.99% (166/437) of the patients with reducible type I fractures in this study. When intraoperative traction reduction is difficult, the proximal fracture segment is locked to the posterior edge of the distal fracture segment according to lateral fluoroscopy, or if the fracture remains locked after more than three repeated reductions, these fractures can be classified as irreducible type II‐2 fractures.

This preoperative prediction is difficult but has clinical significance. Surgical planning for fractures preoperatively classified as reducible type I fractures based on these criteria should prepare for the possibility of the fracture actually being an irreducible type II‐2 fracture.

#### 4.1.3 Irreducible Type II‐3 Fractures and Reducible Type II Fractures

In this study, 22 fractures were classified as irreducible type II‐3 fractures; however, good reduction was achieved with only closed traction for three fractures, which was considered to be related to the preservation of the soft tissue splint. Forty‐seven fractures were classified as reducible type II fractures, and good reduction was achieved with closed traction, likely due to having less severe injury with good preservation of the bony structures and soft tissue splints.

#### 4.1.4 Irreducible Type III Fractures

Irreducible type III fractures were the most prevalent of irreducible intertrochanteric femoral fractures. These fractures are the most difficult to reduce and require increased traction to correct the shortening deformity, resulting in further flexion and external rotation of the proximal fracture segment. In addition, reverse oblique intertrochanteric fractures with lateral wall fractures also have a high rate of internal fixation failure [3133]. Several authors have suggested that the lateral wall should be reinforced simultaneously with intramedullary nailing to reduce the fixation failure rate [31]. According to the new fracture classification criteria, 89 fractures were classified as irreducible type III fractures, 52 of which had clear difficulties of intraoperative fracture reduction, whereas the remaining 37 achieved good reduction with closed traction, which was considered to be related to incomplete destruction of the soft tissue splints (iliofemoral and pubofemoral ligaments).

### 4.2 Relationship Between the New and Traditional (AO/OTA) Classification Systems

The new and traditional (AO/OTA) classification systems are closely related (see Table 5 for details). Irreducible type I fractures are classified as 31A1 fractures with obvious separation displacement in the AO system, and we divided these fractures into four subtypes according to the fracture line between the greater and lesser trochanters (Figure 1a–d). Irreducible type II fractures include several subtypes of 31A2 fractures in the AO system. The AO system classifies 31A2 fractures with one intermediate fracture block between the trochanters as 31A2.1 fractures, 31A2 fractures with multiple intermediate fracture blocks as 31A2.2 fractures, and 31A2 fractures with the fracture line extending more than 1 cm below the lesser trochanter as 31A2.3 fractures. The new classification system does not consider the number of intermediate fracture blocks but rather the degree of injury to the ligaments and joint capsule near the intertrochanteric line. Fractures with a large degree of damage, causing the proximal fragment to embed forward significantly, are classified as irreducible type II‐1 fractures (Figure 1e). A small number of fractures in which the proximal fragments embedded in the medullary cavity of the distal fracture segments were classified as irreducible type II‐2 fractures (Figure 1f). When these fractures are well protected by the soft tissue splint, most of which can be well repositioned by traction, they are first classified as reducible type I fractures. A small number of fractures that cannot be repositioned by repeated traction are classified as irreducible type II‐2 fractures and cannot be predicted by preoperative imaging but rather are diagnosed only after repeated intraoperative traction without good reduction. 31A2.3 fractures (with a concomitant proximal femur fracture) in the AO system are classified in the new system as irreducible type II‐3 fractures(Figure 1g). Irreducible type III fractures are 31A3 fractures in the AO system (Figure 1h–k). Reducible type I fractures are 31A2 fractures (with the proximal fragment embedded in the medullary cavity of the distal fracture segment) in the AO system (Figure 1l), accounting for the majority of 31A2 fractures. Reducible type II fractures, which are 31A1 or 31A2 fractures in the AO system (Figure 1m), have no significant displacement.

**TABLE 5:** Relationship between the new and traditional (AO/OTA) classification systems.

AO system	The new system	
	Irreducible	Reducible
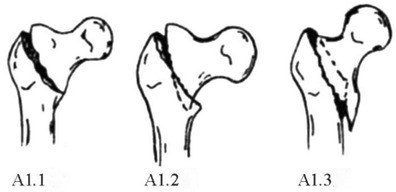 AO Classification: Type 31A1	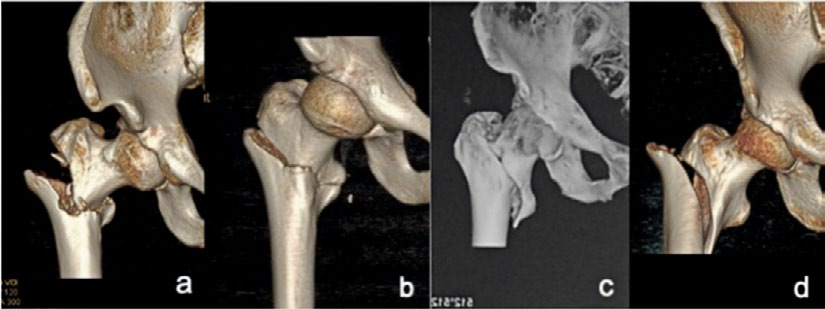 a: Type I‐1; b: Type I‐2; c: Type I‐3; d: Type I‐4 (obvious separation displacement of 31A1)	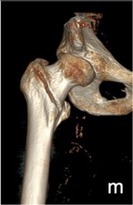 Type II(no obvious separation)
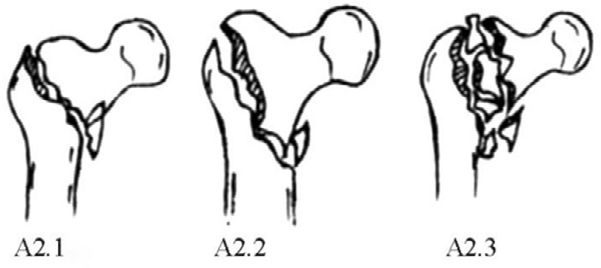 AO Classification: Type 31A2	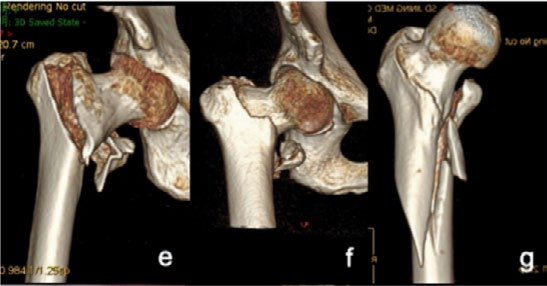 e: Type II‐1; f: Type II‐2*; g: Type II‐3 (Based on the anteroposterior relationship between the proximal and distal fracture segments in the sagittal plane and the presence or absence of concurrent proximal fractures.)	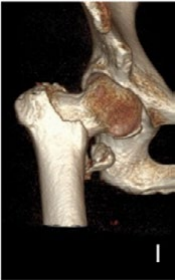 Type I*
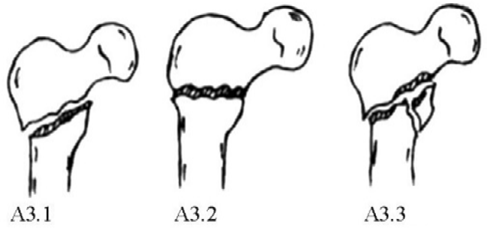 AO Classification: Type 31A3	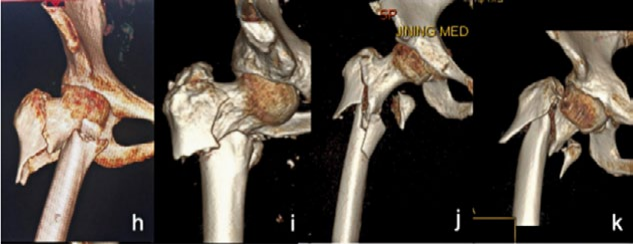 h: Type III‐1; i: Type III‐2; j: Type III‐3; k: Type III‐4 (The new classification is essentially identical to the AO Classification Type 31A3, with the addition of a special subtype involving concomitant femoral neck fracture.)	

* Most fractures of this type can be easily reduced and are classified as Reducible Type I. A small subset that remains difficult to reduce adequately after three attempts at traction on the traction table is categorized as Irreducible Type II.

### 4.3 Analysis of Fracture Classification Accuracy

Analysis of the recorded degree of difficulty of intraoperative reduction among patients within each new fracture classification by the two nonoperating surgeons indicated that the accuracy of the new subtyping was 78.4% overall and 64.71% for irreducible fractures. These results indicate that 64.71% of the fractures predicted to be difficult to reduce according to the new classification criteria were truly difficult to reduce. For these fractures, the anterior approach, a minimally invasive clamp reduction technique, may be chosen [32]. The remaining 35.29% of these fractures could be effectively reduced by closed traction, likely due to the incomplete destruction of the soft tissue hinges. Furthermore, nearly all irreducible fractures can be classified under the new system. This approach will facilitate clinical practice, guiding early preoperative planning, hierarchical diagnosis, and treatment; it also will facilitate preoperative decision‐making and increase patient safety by allowing surgeons to be carefully selected based on predicted surgical difficulty [33]. In this study, using the new classification system, the percentage of intertrochanteric fractures predicted to have difficulty of reduction was 32.76%, which is higher than that previously reported (3%–17%) [2, 4, 6]. Based on previously reported information, only the 31A1 and 31A2 AO‐classified fractures were included in this study; 31A3 fractures were excluded. However, most 31A3 fractures are irreducible [7]. This decision was made after reviewing cases from many hospitals of fractures with difficulty of reduction; thus, the large proportion of irreducible intertrochanteric fractures reported here could suggest data bias.

### 4.4 Follow‐Up Analysis

As shown in Table 4, complications were concentrated mainly in the coxa vara, and patients were left with varying degrees of claudication. Irreducible type III fractures (reverse oblique intertrochanteric fractures) are the most likely to present with complications, followed by irreducible type II‐1 fractures. Therefore, prioritizing these two irreducible types for surgical fixation is important. In conclusion, the new classification system presented here can predict the difficulty of reduction, guide treatment, and predict the prognosis of the fracture.

## 5 Limitations and Strengths

The number of patients in this study was relatively small, and the new fracture classification criteria need to be further refined and supplemented. Furthermore, this classification system requires verification via prospective studies at additional institutions. The accuracy of this system is expected to improve as the operator's proficiency in applying the new fracture classification criteria and understanding their clinical applications improves. In addition, many of the criteria in the new classification system lack quantification, resulting in relatively low accuracy.

This new classification system can predict the difficulty of reduction based on preoperative three‐dimensional (3D) reconstructed CT imaging data. This allows surgeries to be assigned based on predicted difficulty to surgeons with appropriate levels of experience and enables the tailoring of preoperative preparations to the specific fracture type, thereby increasing patient safety.

## 6 Conclusions

In summary, these classification criteria for femoral intertrochanteric fractures, which are based on reducibility or irreducibility, can accurately predict the difficulty of reduction for the vast majority of reducible intertrochanteric femur fractures as well as the majority of irreducible intertrochanteric femur fractures.

## Author Contributions

All the authors participated in the study design, interpretation of the studies, analysis of the data, and review of the manuscript. QJY, ZYF, and ZFH carried out the data analysis and drafted the manuscript. HL conceived the study, participated in its design and coordination, and helped draft the manuscript. XLT and ZQS helped collect the data and perform the statistical analysis. All authors read and approved the final manuscript.

## Ethics Statement

This study was approved by the ethics committee of the Affiliated Hospital of Jining Medical University (IRB No. 2024C037). Since this study is retrospective, uses identifiable imaging data for research purposes, and does not involve personal privacy or commercial interests, the “Waiver of Informed Consent” was approved by the Medical Research Ethics Committee of the Affiliated Hospital of Jining Medical University, as detailed in the Appendix.

## Consent

The authors have nothing to report.

## Conflicts of Interest

The authors declare no conflicts of interest.

## Data Availability

All the datasets analyzed during this study are available from the corresponding author upon reasonable request.

## References

[1] P. Kumar , R. K. Rajnish , S. Sharma , and M. S. Dhillon , “Proximal Femoral Nailing is Superior to Hemiarthroplasty in Ao/Ota A2 and A3 Intertrochanteric Femur Fractures in the Elderly: A Systematic Literature Review and Meta‐Analysis,” International Orthopaedics 44 (2020): 623–633, 10.1007/s00264-019-04351-9. 31201487

[2] H. D. Moehring , G. P. Nowinski , M. W. Chapman , and J. P. Voigtlander , “Irreducible Intertrochanteric Fractures of the Femur,” Clinical Orthopaedics and Related Research 339 (1997): 197–199, 10.1097/00003086-199706000-00027. 9186220

[3] G. Z. Said , O. Farouk , and H. G. Said , “An Irreducible Variant of Intertrochanteric Fractures: A Technique for Open Reduction,” Injury 36 (2005): 871–874, 10.1016/j.injury.2005.01.011. 15949491

[4] G. Sharma , G. N. K. kumar , S. Yadav , et al., “Pertrochanteric Fractures (Ao/Ota 31‐A1 and A2) not Amenable to Closed Reduction: Causes of Irreducibility,” Injury 45 (2014): 1950–1957, 10.1016/j.injury.2014.10.007. 25458060

[5] R. Chandak , N. Malewar , A. Jangle , R. Agarwal , M. Sharma , and A. Kekatpure , “Description of New ‘Epsilon Sign’ and its Significance in Reduction in Highly Unstable Variant of Intertrochanteric Fracture,” European Journal of Orthopaedic Surgery and Traumatology 29 (2019): 1435–1439, 10.1007/s00590-019-02478-4. 31236683

[6] Y. S. Chun , H. Oh , Y. J. Cho , and K. H. Rhyu , “Technique and Early Results of Percutaneous Reduction of Sagittally Unstable Intertrochateric Fractures,” Clinical Orthopaedics and Related Research 3 (2011): 217–224, 10.4055/cios.2011.3.3.217. PMC3162202 21909469

[7] Y. L. Hao , Z. S. Zhang , F. Zhou , et al., “Predictors and Reduction Techniques for Irreducible Reverse Intertrochanteric Fractures,” Chinese Medical Journal 132 (2019): 2534–2542, 10.1097/CM9.0000000000000493. 31658157 PMC6846246

[8] Y. Ikuta , Y. Nagata , and Y. Iwasaki , “Preoperative Radiographic Features of Trochanteric Fractures Irreducible by Closed Reduction,” Injury 50 (2019): 2014–2021, 10.1016/j.injury.2019.06.035. 31327460

[9] D. K. Tong , W. B. Ding , G. C. Wang , et al., “Clinical Classification and Strategies for Irreducible Femur Intertrochanteric Fractures,” Chinese Journal of Orthopaedics and Traumatology 19 (2017): 109–114.

[10] Y. Zhao , F. Zhu , Q. Chang , et al., “Research on the Classification Criteria of Femoral Intertrochanteric Fractures Based on Irreducibility or not,” Zhongguo Xiu Fu Chong Jian Wai Ke Za Zhi 35, no. 9 (2021): 1086–1092, 10.7507/1002-1892.202103233. 34523271 PMC8444130

[11] J. B. Yuan , Z. D. Zhu , X. M. Tang , et al., “Classification and Reduction Strategies for Irreducible Intertrochanteric Femoral Fracture Based on Anatomy,” Chinese Journal of Tissue Engineering Research 9 (2022): 1341–1345, 10.12307/2022.425.

[12] D. K. Tong , W. B. Ding , G. C. Wang , et al., “Classification and Reduction Techniques of Irreducible Intertrochanteric Fractures,” Chinese Journal of Orthopaedics and Traumatology 24 (2021): 238–246.

[13] M. R. Baumgaertner and B. D. Solberg , “Awareness of Tip‐Apex Distance Reduces Failure of Fixation of Trochanteric Fractures of the Hip,” Journal of Bone and Joint Surgery. British Volume (London) 79 (1997): 969–971, 10.1302/0301-620x.79b6.7949. 9393914

[14] G. Kyriakopoulos , A. Panagopoulos , E. Pasiou , et al., “Optimizing Fixation Methods for Stable and Unstable Intertrochanteric Hip Fractures Treated With Sliding Hip Screw or Cephalomedullary Nailing: A Comparative Biomechanical and Finite Element Analysis Study,” Injury 53 (2022): 4072–4085, 10.1016/j.injury.2022.10.006. 36272844

[15] Y. Kim , K. Dheep , J. Lee , et al., “Hook Leverage Technique for Reduction of Intertrochanteric Fracture,” Injury 45 (2014): 1006–1010, 10.1016/j.injury.2014.02.007. 24731692

[16] Y. Zhao , Z. Jiang , T. Li , et al., “Treatment of Irreducible Intertrochanteric Femoral Fracture With Minimally Invasive Clamp Reduction Technique via Anterior Approach,” Zhongguo Xiu Fu Chong Jian Wai Ke Za Zhi 35 (2021): 544–549, 10.7507/1002-1892.202012030. 33998205 PMC8175198

[17] J. W. Yurek , N. A. Doerr , A. Tang , A. S. Kohring , F. A. Liporace , and R. S. Yoon , “Assessing the Necessity of Extra Reduction Aides in Intramedullary Nailing of Intertrochanteric Hip Fractures,” Hip Pelvis 35, no. 3 (2023): 183–192, 10.5371/hp.2023.35.3.183. 37727297 PMC10505845

[18] J. W. Kim , J. I. Yoo , J. T. Kim , W. S. Choy , and Y. Cha , “Clinical and Radiological Characteristics of Lesser Trochanter Splitting Irreducible Intertrochanteric Fractures,” Clinics in Orthopedic Surgery 15, no. 4 (2023): 560–566, 10.4055/cios22325. 37529199 PMC10375813

[19] S. J. Hu , S. M. Chang , S. C. Du , L. Z. Zhang , and W. F. Xiong , “Two‐Part Intertrochanteric Femur Fractures With Bisection of the Lesser Trochanter: An Irreducible Fracture Pattern,” Geriatr Orthop Surg Rehabil 14 (2023), 10.1177/21514593231153827. PMC9880592 36712599

[20] S. Zhang , S. Hu , S. Du , L. Z. Zhang , and W. F. Xiong , “Two‐Part Pertrochanteric Femur Fractures With Bisection of the Lesser Trochanter: An Irreducible Fracture Pattern With Soft Tissue Interposition,” Journal of Tongji University (Medical Science) 41 (2020): 772–778.

[21] H. Hu , G. Chen , X. Wu , M. Lin , and H. Lin , “Reduction With Pre‐Drilling Combined With A Finger Reduction Tool in Difficult‐to‐Reduce Intertrochanteric Fracture,” Orthopaedic Surgery 14 (2022): 2750–2756, 10.1111/os.13447. 36056594 PMC9531095

[22] Q. Fang , J. Han , W. Liu , D. Wang , Z. Ge , and G. Wang , “Predictors of and Predictive Nomogram for Cut‐Out of Proximal Femur Nail Anti‐Rotation Device in Intertrochanteric Fractures,” Archives of Orthopaedic and Trauma Surgery 143, no. 7 (2023): 3985–3995, 10.1007/s00402-022-04676-y. 36348087

[23] X. W. Huang , G. Q. Hong , Q. Zuo , and Q. Chen , “Intracortical Screw Insertion Plus Limited Open Reduction in Treating Type 31‐A3 Irreducible Intertrochanteric Fractures in Elderly Individuals,” World Journal of Clinical Cases 9 (2021): 9752–9761, 10.12998/wjcc.v9.i32.9752. 34877314 PMC8610916

[24] K. B. Fang , X. C. Lin , S. J. Shi , and Z. S. Dai , “Treatment of Irreducible Femoral Intertrochanteric Fractures Using A Wire‐Guided Device,” Chinese Journal of Traumatology 24 (2021): 104–108, 10.1016/j.cjtee.2021.01.002. 33549392 PMC8071721

[25] X. W. Huang , G. Q. Hong , J. Fan , X. Li , and Q. Chen , “Clinical Efficacy of Fixation Bone Fragments by Screw Though Cortical Bone Tunnel Combined With Limited Open Reduction in Treating 31‐A3 Type Irreducible Femoral Intertrochanteric Fractures in Elderly People,” Zhonghua Yi Xue Za Zhi 100 (2020): 674–678. 32187910 10.3760/cma.j.issn.0376-2491.2020.09.006

[26] T. Jiang , H. Gao , T. Liu , et al., “Observation of the Clinical Efficacy of Percutaneous Reduction by Leverage Combined With Intramedullary Nail Internal Fixation in the Treatment of Irreducible Femoral Intertrochanteric Fracture: A Retrospective Single‐Arm Cohort Study,” Ann Transl Med 10 (2022): 822, 10.21037/atm-22-2846. 36034997 PMC9403922

[27] Q. Huang , Y. Xu , H. Xue , et al., “Percutaneous Reduction With Double Screwdrivers Versus Limited Open Reduction in the Treatment of Irreducible Extracapsular Hip Fractures,” BMC Musculoskeletal Disorders 23 (2022): 429, 10.1186/s12891-022-05390-x. 35524242 PMC9077818

[28] W. Zhang , R. P. Antony Xavier , J. Decruz , Y. D. Chen , and D. H. Park , “Risk Factors for Mechanical Failure of Intertrochanteric Fractures After Fixation With Proximal Femoral Nail Antirotation (Pfna Ii): A Study in a Southeast Asian Population,” Archives of Orthopaedic and Trauma Surgery 141 (2021): 569–575, 10.1007/s00402-020-03399-2. 32296964

[29] P. Karayiannis and A. James , “The Impact of Cerclage Cabling on Unstable Intertrochanteric and Subtrochanteric Femoral Fractures: A Retrospective Review of 465 Patients,” European Journal of Trauma and Emergency Surgery 46 (2020): 969–975, 10.1007/s00068-018-01071-4. 30612147

[30] K. Buyukdogan , O. Caglar , S. Isik , M. Tokgozoglu , and B. Atilla , “Risk Factors for Cut‐Out of Double Lag Screw Fixation in Proximal Femoral Fractures,” Injury 48 (2017): 414–418, 10.1016/j.injury.2016.11.018. 27889112

[31] A. Gupta , H. Bansal , A. Kumar , S. Mittal , and V. Trikha , “The Effect on Outcomes of the Application of Circumferential Cerclage Cable Following Intramedullary Nailing in Reverse Intertrochanteric Femoral Fractures,” European Journal of Orthopaedic Surgery and Traumatology 30 (2020): 949, 10.1007/s00590-020-02641-2. 32152746

[32] J. Qiu , Z. Jiang , L. Han , et al., “Treatment of Irreducible Intertrochanteric Femoral Fracture With a Minimally Invasive Clamp Reduction Technique via the Anterior Approach,” Journal of Orthopaedic Surgery and Research 18 (2023): 167, 10.1186/s13018-023-03641-8. 36871013 PMC9985279

[33] J. Singh , P. Gupta , S. Kanwar , and A. Pookunju , “Ipsilateral Intertrochanteric Fracture With Posterior Dislocation of Hip,” Journal of Orthopaedic Case Reports 13, no. 11 (2023): 142–146, 10.13107/jocr.2023.v13.i11.4038. PMC10664238 38025348

